# Transcriptional and Metabolic Changes Associated to the Infection by *Fusarium verticillioides* in Maize Inbreds with Contrasting Ear Rot Resistance

**DOI:** 10.1371/journal.pone.0061580

**Published:** 2013-04-18

**Authors:** Valeria A. Campos-Bermudez, Carolina M. Fauguel, Marcos A. Tronconi, Paula Casati, Daniel A. Presello, Carlos S. Andreo

**Affiliations:** 1 Centro de Estudios Fotosintéticos y Bioquímicos, Facultad de Ciencias Bioquímicas y Farmacéuticas, Universidad Nacional de Rosario, Rosario, Argentina; 2 Instituto Nacional de Tecnología Agropecuaria, Pergamino, Argentina; TGen, United States of America

## Abstract

*Fusarium verticillioides* causes ear rot and grain mycotoxins in maize (Zea mays L.), which are harmful to human and animal health. Breeding and growing less susceptible plant genotypes is one alternative to reduce these detrimental effects. A better understanding of the resistance mechanisms would facilitate the implementation of strategic molecular agriculture to breeding of resistant germplasm. Our aim was to identify genes and metabolites that may be related to the *Fusarium* reaction in a resistant (L4637) and a susceptible (L4674) inbred. Gene expression data were obtained from microarray hybridizations in inoculated and non-inoculated kernels from both inbreds. Fungal inoculation did not produce considerable changes in gene expression and metabolites in L4637. Defense-related genes changed in L4674 kernels, responding specifically to the pathogen infection. These results indicate that L4637 resistance may be mainly due to constitutive defense mechanisms preventing fungal infection. These mechanisms seem to be poorly expressed in L4674; and despite the inoculation activate a defense response; this is not enough to prevent the disease progress in this susceptible line. Through this study, a global view of differential genes expressed and metabolites accumulated during resistance and susceptibility to *F. verticillioides* inoculation has been obtained, giving additional information about the mechanisms and pathways conferring resistance to this important disease in maize.

## Introduction

*Fusarium verticillioides*, (Saccardo) Nirenberg [( = *F. moniliforme* (Sheldon), teleomorph *Gibberella moniliformis* (Wineland) ( = *G. fujikuroi* (Sawada Ito in Ito & Kimura, mating population A)] is the prevalent kernel and ear rotting pathogen in Argentina [1]. This fungus produces yield losses [2] and grain contamination with several mycotoxins, including fumonisins [3]–[5]. Fumonisin occurrence in foods and feeds has wide economic implications [6], and the development of management strategies for controlling *F. verticilliodes* infection and fumonisin contamination are needed to reduce detrimental effects on human and animal health [7]. There is a positive association between visible symptoms caused by *F. verticillioides* infection and mycotoxin concentration [8], [9]. *F. verticillioides* infection and fumonisin contamination occur as maize kernels come up to physiological maturity, and increase during the season up to the average harvest date [10], [11]. *F. verticillioides* enters the ear through the silk channel, spreads within the ear on the silks, and infects isolated single kernels or groups of kernels in localized areas of the ear [12]. After conidia reach maize silks, penetration and colonization occur with a series of biochemical reactions being affected by resistance mechanisms in the host plant. Therefore, developing and using resistant hybrids may prevent both ear rot progress and grain fumonisin contamination. Although genetic variation for resistance to *Fusarium* ear rot exists among inbred lines and hybrids in field maize [13]–[15], there is no evidence of complete resistance to either ear rot or fumonisin accumulation. High levels of disease resistance were observed in Argentinean landraces [13] that are being used to improve elite germplasm. In part, the difficulty in developing more resistant genotypes is due to the lack of understanding of the factors important to *F. verticillioides* infection and fumonisin accumulation.

Plants defend themselves against pathogen attack by activating a multicomponent defense response. Activation of signal transduction network after pathogen recognition results in a reprogramming of cellular metabolism involving large changes in gene activity [16]. Expressions of a large array of genes whose products are involved in diverse primary and secondary metabolic pathways are rapidly induced or strongly up-regulated [17]. These responses include induction of pathogenesis related (PR) genes like those coding for glucanases and chitinases, production of secondary metabolites or reinforcement of cell walls. The identification of genes controlling resistance to this fungus in ear rot would facilitate their introgression into commercial hybrids. In plant-pathogen interactions, microarray studies provide a more comprehensive understanding of molecular responses in the infection process, allowing the elucidation of mechanisms involved in resistance. The aim of this work was to identify gene transcripts and metabolic host factors that could control plant resistance and susceptibility to *F. verticillioides* infection in maize. The knowledge accumulated in these studies will serve as fundamental basis to the development of original strategic agriculture.

## Results

### Symptom Severity, Grain Ergosterol and Fumonisin Content

For all the experiments, two maize inbreds with contrasting phenotypes were chosen. Inbred L4637 was classified as resistant and L4674 as susceptible according to their field behavior after a fungal inoculation treatment in a previous experiment [14], [18], [19]. *F. verticillioides* inoculation was carried out through the silk channel, considering that it is the principal entry route of this fungal pathogen. Disease severity, ergosterol and fumonisin contents were tested in the grains to evaluate field responses of the two maize selected lines (Table 1).

**Table 1:** Ear rot severity, ergosterol and fumonisin concentration in grains of two inbred subjected to artificial inoculation with conidial suspensions of *F. verticillioides.*

Inbred	Fumonisin concentration (ppm)	Disease severity (%)^1^	Ergosterol concentration (ppm)
L4637	144,4	2,22*	4,41*
L4674	406,6	81,71	48,37

1 Percentage of the ear visibly covered with mold after inoculation with *F. verticillioides*.

* Differences between means are significant at p<0.05.

**Figure 1:**
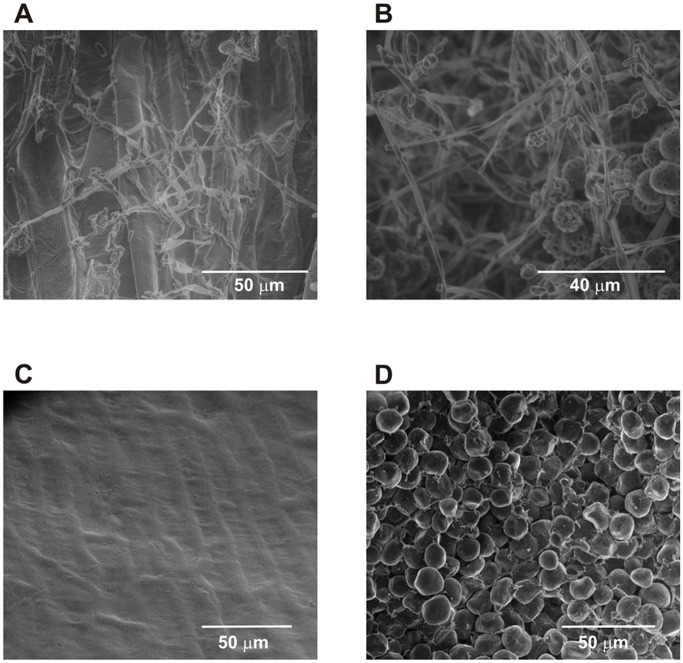
Scanning electron micrographs of *Fusarium verticillioides* inoculated maize kernels of L4637 (resistant) and L4674 (susceptible) inbreds. Hyphae growing at the pericarp surface (A) and endosperm (B) in susceptible inbred. Absence of external (C) and internal (D) fungal infection in the resistant inbred.

The resistant inbred exhibited lower disease severity and grain fumonisin accumulation compared to the susceptible one (Table 1). Disease severity only determines the visual damage in grains; therefore, to analyze the fungus content in the sample, we measured ergosterol levels in infected tissues. Ergosterol is a specific component of the fungal membrane and its analysis is commonly used to estimate the fungal biomass formed on natural solid substrates [20]. After inoculation, L4637 exhibited lower grain fumonisin, ergosterol concentration and disease severity compared to L4674 (Table 1), suggesting that kernels of the resistant inbred are less likely to be invaded by the fungi. This is supported by the absence of the pathogen in the pericarp surface and internal structures in L4637 inoculated kernels analyzed by scanning electronic microscopy (Figure 1).

### Microarray Analysis of Transcriptomic Changes in Kernels Under F. Verticillioides Infection

The global gene expression profiles of maize kernels under *F. verticillioides* infection were examined using Maize Oligonucleotide Arrays to measure and compare the accumulation of transcripts of more than 30,000 maize genes. In total, we identified 510 differentially expressed sequences when the analysis was done using samples from the L4674 inbred. Among these, 79 have not assigned functions. From the assigned genes, we detected 293 up-regulated and 138 down-regulated genes. In the same comparison using samples from the L4637 inbred, only 37 sequences were differentially expressed, 32 of these genes have assigned putative functions. Among these, 12 were up-regulated and 20 down-regulated. All of the differentially expressed genes are reported in File S1 with the corresponding p-values.

The differentially expressed sequences were classified into 36 functional categories using the MapMan software [21], which relied on its own ontology to classify genes and metabolites, and provides a modular system to visualize the results in the context of pathways and processes. As the functional annotation of maize sequences is still limited, the functional classification implemented in the mapping files was that of the Oryza sativa spp. Japonica genes. In general, we observed that the majority of transcripts were classified in protein, RNA, DNA, stress, transport, signaling and cell metabolism categories. However, some groups had lower numeric relevance in our samples.

**Figure 2:**
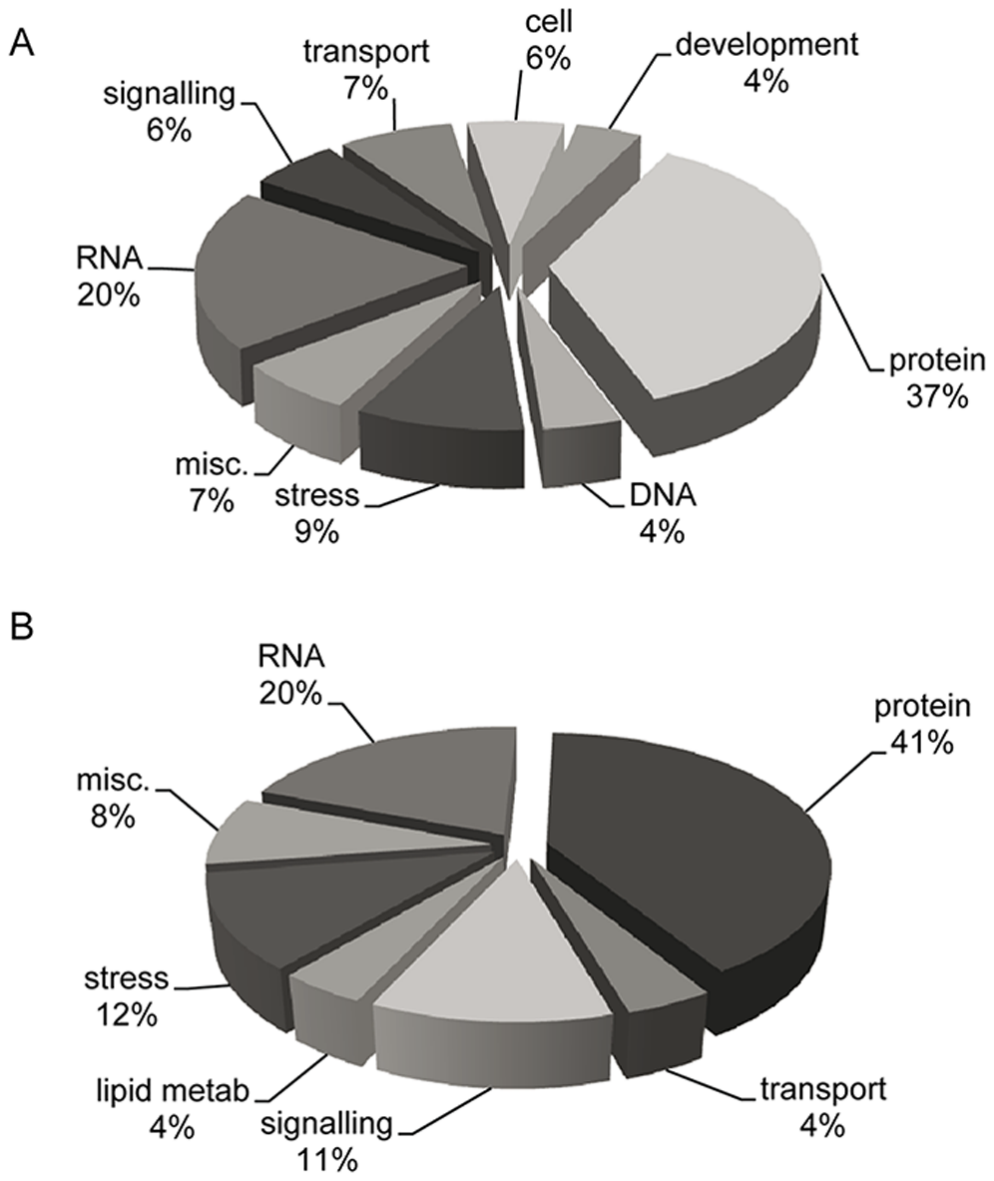
Comparison of gene expression in grain tissues of L4637 (A) and L4674 (B) maize inbreds after *Fusarium verticillioides* infection.

When the transcriptomes from inoculated vs non inoculated samples from L4674 were compared, we found that among the annotated genes, those belonging to protein, RNA, and stress categories represented the most important fungal inoculation responsive genes, accounting for around 70 % of transcript changes, whereas those classified in transport, signaling, cell metabolism, miscellaneous and DNA metabolism were also affected but in less proportion (Figure. 2A). The analysis of expression in each category reveals a greater number of induced genes after the fungal inoculation, where the RNA, protein and cell metabolism categories show the most significant effects. Interestingly, the transcript with higher induction after inoculation corresponds to a zein protein, with a log_2_ fold change up to 4.5, which was not included in the MapMan analysis as it does not show homology to any transcript in rice (File S1). In the list of up-regulated genes, there are stress response related transcripts, such as heat shock proteins (MZ00019093), a cellulose synthase-like protein (MZ00021213), a mannitol 1-phosphate dehydrogenase (MZ00022515), a sucrose synthase (MZ00026383) and an endoxyloglucan transferase (MZ00054823), signal transduction like a chromatin-remodeling factor CHD3 (MZ00016410), an auxin-independent growth mRNA (axi1) (MZ00029605), a WRKY 70 transcription factor (MZ00054846), a putative Myb-like DNA-binding protein (MZ00018761) and a glycine-rich protein (MZ00016231). The down-regulated genes primarily related to the functions of metabolism and defense, including stress-responsive genes such as a chitinase (MZ00043559), a senescence-associated protein (MZ00035828), a cytochrome P450 (MZ00047108), an auxin transport protein (MZ00036454) and a sugar transport protein (MZ00004123).

For L4637 grain samples, we found that there were only a few transcripts which levels were affected by the inoculation. However, significant changes were observed in transcripts corresponding to the protein, RNA, and stress functional categories. Interestingly, transcript changes correspond to repression in gene expression after fungal inoculation; particularly in the protein category (Figure 2B). However, some transcripts like those encoding a mannitol 1-phosphate dehydrogenase (MZ00022515), an early drought induced protein (MZ00039626) and a physical impedance induced protein (MZ00041473) were up-regulated (File S1).

The transcriptomes of L4637 and L4674 grain samples were also compared. First, non inoculated kernel mRNA samples from both inbreds were compared. From this comparison, it is interesting that L4637 presented higher expression levels of 408 genes in comparison with L4674, many of them encode proteins that participate in resistance responses to pathogens; therefore, it is possible that the resistance observed in this line is due to a preformed or a constitutively expressed defense system. Among the transcripts showing increased levels in the resistant inbred, we found a beta-glucosidase (MZ00044454), metallothioneines (MZ00042895), a member of the 26S proteasome (MZ00041312), a beta-1,3-glucanase (MZ00021961), an arabinoxylan arabinofuranohydrolase isoenzyme AXAH-II (MZ00014360), a transcription factor of WRKY family (MZ00054846), a DNA binding protein (MZ00023051), a phenylalanine ammonia-lyase (MZ00025088) and a lipase (MZ00037469). The most important down-regulated genes encountered were those related to a lipid transfer protein (MZ00003835), a xylanase inhibitor (MZ00014433) and a cytochrome P450 (MZ00035752).

A similar comparison using inoculated grain samples from both inbreds revealed that 204 genes showed increased levels in the L4637. Genes with increased expression corresponded to a beta-glucosidase (MZ00044454), a beta-1,3-glucanase (MZ00027418), a pectinesterase (MZ00030453) and a member of the 26S proteasome (MZ00041312), and the down-regulated genes include a lipid transfer protein (MZ00003835), zein-alpha precursors, a glutathione S-transferase (MZ00015071) and a subtilisin/chymotrypsin inhibitor (MZ00041005).

### Gene Expression Analyses by Real-time Quantitative RT-PCR (qRT-PCR)

**Figure 3:**
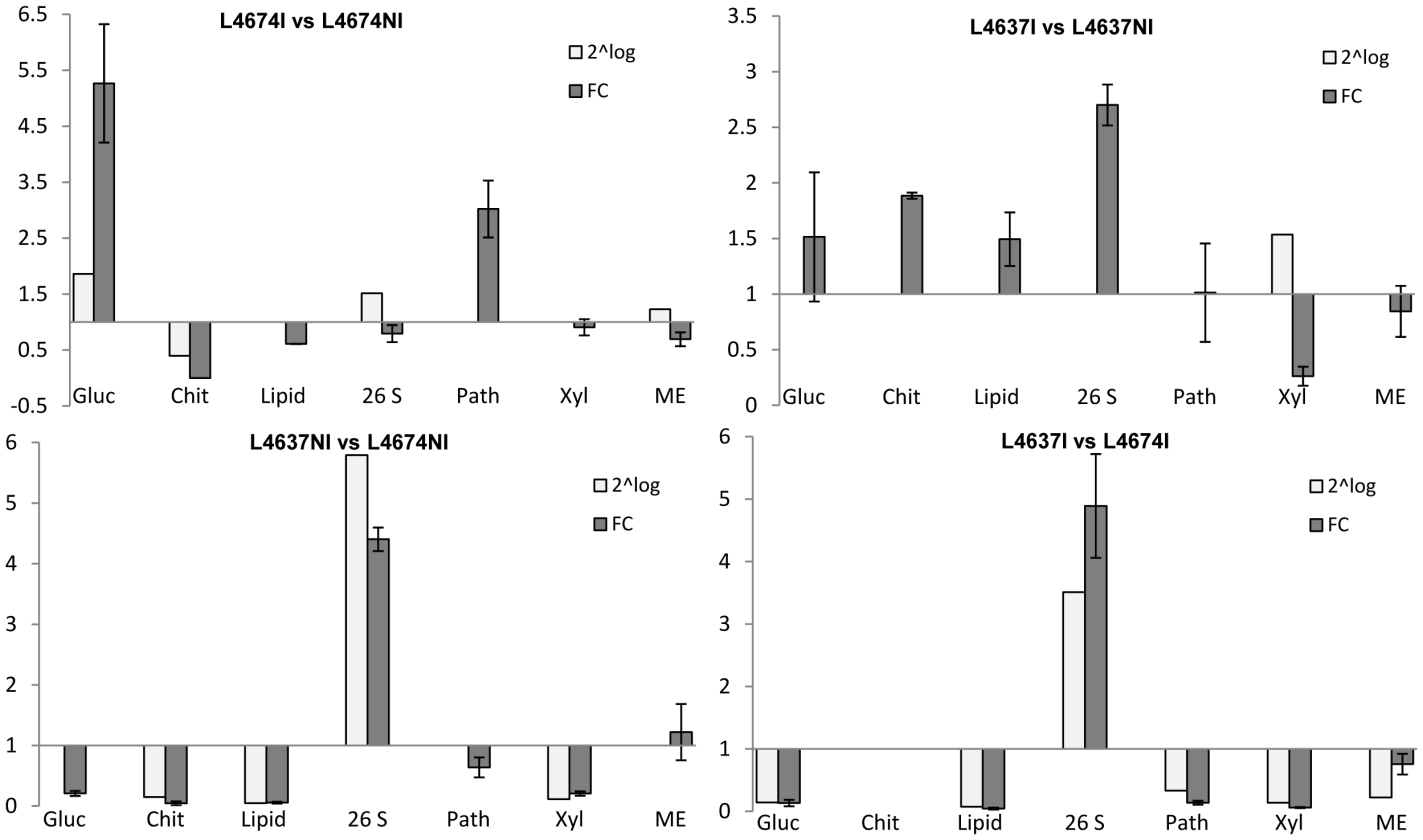
Gene expression analyses by qRT-PCR of seven selected genes. Gluc: *glucanase*, chit: *chitinase*, Lipid: *Nonspecific lipid-transfer protein*, 26S: *26S proteosome*, Path: *pathogen related protein*, Xyl: *xylanase inhibitor*, ME: *malic enzyme,* FC: fold change gene expression, 2^∧^log: antilog_2_ (log_2_ of each comparison of microarrays data).Changes of Defense Genes in Silks.

We further used real-time quantitative RT-PCR to confirm the expression changes of transcripts identified by the microarray analysis. Seven genes were selected and three independent biological replicates were performed. The results show that, despite differences existing between transcripts levels determined by both techniques, mRNA levels of the seven genes analyzed by qRT-PCR correlate well with the microarray results (Figure 3).

**Figure 4:**
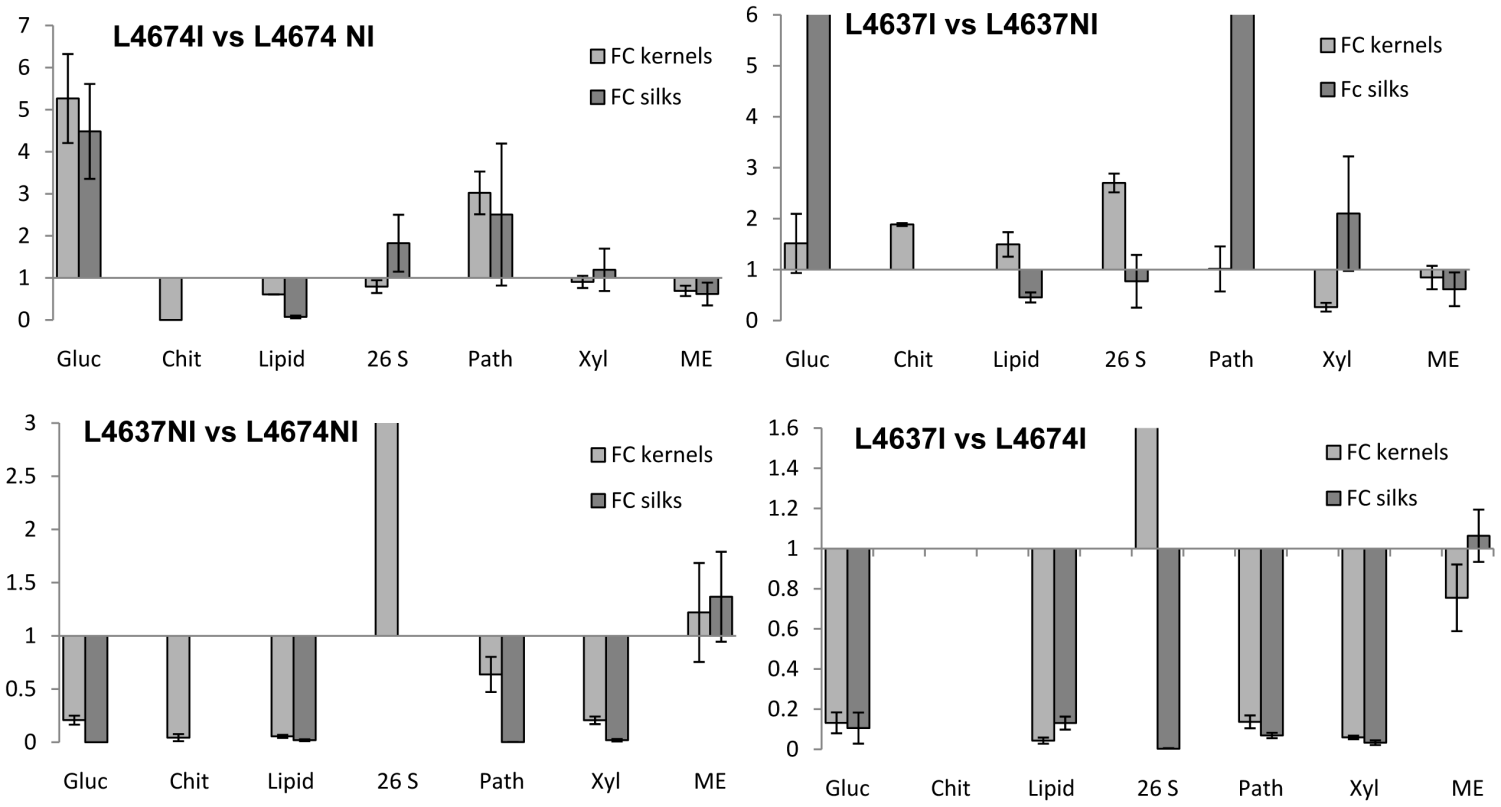
Silk gene expression analyses by qRT-PCR of seven selected genes. Gluc: *glucanase*, chit: *chitinase*, Lipid: *Nonspecific lipid-transfer protein*, 26S: *26S proteosome*, Path: *pathogen related protein*, Xyl: *xylanase inhibitor*, ME: *malic enzyme,* FC: fold change gene expression.

It is known that the most important pathway for ear infection is through silks, causing infection up to 84% of the kernel set [22]. Germinated spores penetrate silks and the mycelium progressively colonizes silk tissues from the ear top to the base. The entire length of the majority of the silks of an ear is infected only after 28 days [23]. Additionally, Lanubile et al. [24] reported that the presence of the fungus could not be detected in silks before 72 h after infection. Based on these statements, we measured the expression levels of genes selected to validate the microarrays experiment by qRT-PCR in silks samples three days after the inoculation. In general, we observed a similar expression pattern to that measured in kernels with some differences (Figure 4). In particular, for the *Glucanase* transcript, fungal inoculation increased the expression of this gene in silks of the susceptible line, as it was also measured in kernels. However, the most notable difference was observed in L4637 silks after inoculation, where the increase in expression of this transcript was considerably higher than that observed in kernels. For the *Chitinase* transcript, we did not observe expression of this gene in silks using the same set of primers used to validate its expression in kernels, indicating that this gene is kernel specific, or at least is not expressed in silks. Although kernel basal expression of this transcript was higher in L4674 than in the L4637 (Figure 4), its expression decreased in L4674 and remained the same in L4637 after inoculation. Basal gene expression of the *Nonspecific lipid-transfer protein AKCS9* in silks was higher in L4674 than in L4637, and inoculation produced a repression of this transcript in both inbreds.

After the inoculation treatment, transcripts for the *26S proteasome non-ATPase regulatory subunit 8* were higher expressed in silks from L4674. However, opposite behavior was observed in silks and kernels of the L4637 inbred. For the *Pathogenesis-related protein 10* transcript, higher basal expression was detected in silks of L4674 compared with L4637; and inoculation increased the expression of this transcript in both inbreds, with higher levels of expression in L4637. The inoculation treatment induced an increase in the expression of the gene encoding the *Xylanase inhibitor protein 1* in silks and kernels of L4637, with no changes in L4674. Finally, the *Malic enzyme* transcript showed lower expression in silks after inoculation, with no differences between inbreds. However, higher expression was detected in kernel tissues of L4674.

### Metabolites Profile Analysis after Fusarium Inoculation in Kernels

In addition to the transcriptome changes analyzed after inoculation with *F. verticillioides* in grains from the resistant and susceptible inbreds, we pursued a metabolomic based approach to study metabolic changes involved in resistance to this pathogen. The identified metabolites were classified into 5 categories according to their functions: sugars, polyols, aminoacids, acids, and polyamins.

Comparisons of sugar levels in *F. verticillioides*-infected and non infected maize kernels (Table 2) revealed that L4637 had higher basal levels of glucose, fructose, galactose and sucrose compared with those of L4674. Additionally, sugar concentration decreased only after inoculation in the susceptible inbred. Conversely, a decrease on turanose levels was observed in both inbreds after inoculation. On the other hand, maltose levels increased greatly after inoculation only in the susceptible inbred.

**Table 2:** Metabolite concentration in grain tissues of L4637 and L4674 maize inbreds under *Fusarium verticillioides* inoculation.

Metabolite*		L4637 NI	L4637 I	L4674 NI	L4674 I
**SUGARS**	cetoglucose	0,07*^a^*	0,05*^a^*	0,38*^a^*	0,34*^a^*
	D-fructose	0,03*^a^*	0,04*^a^*	6,41*^c^*	1,06*^b^*
	glucose	0,07*^a^*	0,45*^a^*	9,90*^c^*	1,92*^b^*
	D-ribose	0,01*^a^*	0,07*^b^*	1,19*^d^*	0,26*^c^*
	sucrose	132,75*^b^*	90,98*^ab^*	202,33*^c^*	53,54*^a^*
	maltose	0,00*^a^*	0,07*^a^*	0,00*^a^*	29,27*^b^*
	turanose	8,36*^c^*	2,62*^b^*	1,56*^b^*	0,11*^a^*
	galactose	0,00*^a^*	0,03*^a^*	1,53*^c^*	0,43*^b^*
**ALCOHOLS**	glycerol	0,04*^a^*	0,29*^b^*	0,41*^c^*	1,17*^d^*
	1-amino-2-propanol	0,01*^a^*	0,01*^a^*	0,05*^c^*	0,04*^b^*
	mannitol	0,00*^a^*	0,02*^a^*	0,04*^a^*	3,17*^b^*
	myo-inositol	0,32*^c^*	0,22*^b^*	0,57*^d^*	0,17*^a^*
	sorbitol	0,00*^a^*	0,03*^a^*	0,06*^a^*	3,48*^b^*
	arabitol	0,02*^a^*	0,03*^a^*	4,21*^c^*	0,71*^b^*
**AMINOACIDS**	L-alanine	0,04*^a^*	0,04*^a^*	0,23*^b^*	0,09*^a^*
	L-proline	0,00*^a^*	0,00*^a^*	0,06*^c^*	0,02*^b^*
	L-asparragine	0,07*^a^*	0,10*^a^*	0,16*^b^*	0,06*^a^*
**ACIDS**	butanoic acid	0,01*^a^*	0,01*^a^*	0,05*^b^*	0,02*^ab^*
	malic acid	0,00*^a^*	0,00*^a^*	0,35*^c^*	0,05*^b^*
	aspartic acid	0,10*^a^*	0,08*^a^*	0,31*^c^*	0,19*^b^*
	1,2,3, tricarboxilic acid	0,00*^a^*	0,02*^a^*	0,55*^c^*	0,37*^b^*
	hexadecanoic acid	0,17*^b^*	0,06*^a^*	0,07*^ab^*	0,05*^a^*
	benzoic acid	0,07*^a^*	0,04*^a^*	0,04*^a^*	0,03*^a^*
	galacturonic acid	0,00*^a^*	0,02*^b^*	0,34*^d^*	0,11*^c^*
**PHOSPHATE**	phosphate	0,05*^a^*	0,11*^a^*	1,44*^b^*	2,11*^c^*
**POLYAMINES**	cadaverine	0,00*^a^*	0,00*^a^*	0,32*^b^*	0,35*^c^*
	putrescine	0,06*^a^*	0,08*^a^*	0,13*^b^*	0,14*^b^*

* For each metabolite different letters means significant differences (p<0,05).

We observed a notable increase in mannitol and sorbitol polyols after inoculation in L4674 but not in L4637. On the other hand, myo-inositol decreased post inoculation but glycerol had the opposite effect in both inbreds. In not inoculated kernels, the amount of arabitol was higher in L4674 than in L4637, and this compound level decreased greatly after inoculation.

Asparagine, alanine and proline were the main amino acids detected in non-inoculated maize kernels, with a significant higher content in L4674. After inoculation, amino acid levels were not changed in L4637 but decreased significatively in L4674. Most significant changes were observed in L4674, where the basal levels of the organic acids were higher than those in L4637, and went down after inoculation. Regarding to polyamin compounds, we only identified cadaverin and putrescin, with increased levels of cadaverin after inoculation in the susceptible line.

## Discussion

In order to extend our knowledge about the biochemical and molecular changes produced by the inoculation with one of the most important maize pathogens, *F. verticillioides*, we evaluated the differential gene expression in moderately resistant and susceptible maize inbreds. We sampled silk and kernel tissues at a late period of *Fusarium* infection in order to assess changes in gene expression and concentration of biochemical compounds after most of the infection process was completed. Thus, some of the mechanisms affecting disease resistance in earlier stages, which were analyzed in previous studies [24]–[26], could have left no remaining molecular or biochemical signals to be detected. Our study focused in all those changes occurring during disease development that remained detectable at the end of the process.

L4637 exhibited lower levels of disease severity, ergosterol and fumonisin concentration compared to L4674, confirming results observed in previous studies [18], [19]. Despite the complexity of the genotypic and environmental effects, both inbreds exhibited different gene expression patterns in response to *F. verticillioides* infection. These findings indicate that there are general maize host plant-pathogen recognition and interaction processes underlying resistance and susceptibility.

While inoculation affected the expression of an important number of genes in L4674, changes observed in L4637 where minor compared to those of the susceptible inbred. No differences between inbreds in the expression of those genes were observed in the non-inoculated treatment. Therefore, these changes could be attributed to specific responses to pathogen infection. The fact that *Fusarium* infection seemed to turn on some genes in the susceptible inbred only, in which kernels were massively colonized, suggests that while L4674 produced substances promoting fungal growth, L4637 may have constitutive mechanisms preventing expression of those genes and fungal colonization. Our results differ from those obtained by Guangsheng and colleagues [26] who found different transcriptional changes in pathogen-affected maize bract tissues, with a significantly higher number of up-regulated genes in the resistant line and only seven up-regulated genes in the susceptible line after Fusarium infection, probably due to the constitutive expression of many defense genes at higher or lower levels than in the resistant line prior to the Fusarium infection.

*Fusarium* induced genes include those for carbohydrate-metabolism-related proteins, detoxification enzymes, lipid-transfer proteins, ribosomal proteins, signal transduction and transcription factors. One of most representative changes was the increment of expressions for α Zein genes after inoculation in L4674. Higher expression of these storage proteins may be a reaction of L4674 after fungal invasion, since changes in endosperm texture were reported to avoid pathogen dissemination [27]. Some other genes that were specifically induced by inoculation were a glycin rich protein (MZ00016231), a Myb_like DNA binding protein (MZ00018761), a putative cellulose synthase-like protein OsCslE2 (MZ00021213), a mannitol 1-phosphate dehydrogenase (MZ00022515) and sucrose synthase 3 (MZ00026383) (File S1).

On the other hand, some genes were also up- regulated in the L4637 vs. L4674 NI comparison, indicating that the resistant line has a preformed defense mechanism to circumvent different stresses. Although the level of transcripts encoding heat shock proteins (Hsp) was significantly increased by infection of *F. verticillioides* in the susceptible inbred, the L4637 resistant line had high constitutive expression of these proteins. The synthesis of such proteins has been reported to increase after various forms of abiotic and biotic stresses [28], [29]. WRKY genes were significantly up-regulated in L4674 after inoculation; however, some of them present higher basal levels in the resistant line compared to the susceptible one. WRKY proteins are a superfamily of transcription factors involved in the signal transduction pathway that are induced early in pathogen attack, and recognizes the W-box of promoters of a large number of defence-related genes [30].

Taking into account that the preferential entry via of *Fusarium* is through maize silks, we analyzed the expression profile of a subset of selected genes in this tissue of fungal inoculated and non inoculated plants using the same primers used to validate the microarray experiments. In our work, an increase in glucanase, 26S proteasome, a pathogenesis related protein and xylanase genes expression were observed after inoculation, both in kernels and silks of the resistant and susceptible lines. These results are according with proposed roles for these genes in pathogen defense [31]–[39]. Surprisingly, although gene expression of a putative chitinase could not be detected in silks, down regulation of its expression occurred after inoculation in kernels of the susceptible line, similarly to what was observed for a lipid transfer protein in silks of both the susceptible and the resistant line. However, down regulation of defense genes has been reported as part of the susceptible interaction to reduce host-based defense responses that impeded pathogen growth that led to rapid pathogen development and subsequent colonization of host tissues [40].

To extend our knowledge about the interaction in this pathosystem, the metabolomic profile of resistant and susceptible maize kernels was analyzed. Although almost no changes were observed in the resistant line as result of fungal inoculation, significant variations were detected in the susceptible line. In particular, we observed that major changes occur in pathways related to carbon and amino acid metabolism, as well as in polyamine accumulation in the susceptible line.

Sucrose and its cleavage products glucose and fructose are central molecules for metabolism and sensing in higher plants. Rapid mobilization of these carbohydrates seems to be an important factor determining the outcome of plant-pathogen interactions. In particular, in source cells, reprogramming of the carbon flow from sucrose to hexoses may be a crucial process during defense [41], evidenced by induction of cell wall and vacuolar invertase activity. In addition, the pathogen tries to manipulate plant carbohydrate metabolism for its own need, the increase in invertase expression could also be viewed as part of fungal pathogenesis, because fungi use glucose rather than sucrose. In the present study, a decrease in sucrose levels in the susceptible line after fungal inoculation could hence be related to an induction of invertase (MZ00026418) and sucrose synthase (MZ00026383), corroborated by transcript expression observed in microarrays experiments.

Plant soluble sugars (glucose, fructose and sucrose) could rapidly be metabolized and be converted to fungal metabolites (mannitol, trehalose and glycogen) [42]. Sequestering of host fructose as mannitol, depends on the fact that only a small number of plants have the ability to metabolize mannitol, and the fungus becomes a carbohydrate sink within the host [43]. Differential ability of the fungus to colonize L4637 and L4674 could explain the increase in polyols observed only in the susceptible line. The increment in mannitol 1-phosphate dehydrogenase expression observed in microarray analysis could explain in part the differences in mannitol levels in metabolite analysis.

Aminoacids could also be consumed during fungal colonization and would act as a nitrogen sink during infection [42]. Decrease in aminoacids levels in kernels of L4674 could be related with the ability of *F. verticilliodes* to preferentially colonize this line.

Polyamines are well known as metabolites rapidly induced by diverse biotic and abiotic stresses in plants. Gardiner et al. [44] described a preliminary experiment that showed that putrescine was elevated in wheat heads during *Fusarium* disease development, and hypothesised that the pathogen may perceive polyamines and related amino acids as cues for the production of toxins during the infection process in wheat. In our study, no changes in polyamins content were observed in the resistant inbred after inoculation. Although no changes were detected in the susceptible line after inoculation, elevated basal levels of cadaverin and putrescin were encounted in non-inoculated sample. This fact may stimulate micotoxin production once the pathogen invade this line.

In summary, inoculation with *F. verticillioides* caused no important changes in transcriptional and metabolomic profiles detected in the resistant line compared with susceptible inbred, suggesting that a preformed or constitutive defense mechanism may confer L4637 an advantage against *F. verticillioides* infection. The research presented here represents a combination of biochemical and molecular approaches and provide insights to understand the metabolic and transcriptional changes following maize infection with *F. verticillioides* and will likely have relevance to other pathosystems.

## Materials and Methods

### Ethics Statements

No specific permits were required for the described field studies. This land is owned by the Instituto Nacional de Tecnología Agropecuaria (INTA). This Institution counts with several hectares for field assays. People from this institution are allowed to use this area for experimental purpose. CMF and DAP are stable personal of INTA. We confirm that the location is not privately-owned or protected in any way. We confirm that the field studies did not involve endangered or protected species.

### Plant Material and Field Assay

Two maize inbreds that had exhibited moderate resistance (L4637) and susceptibility (L4674) to *Fusarium* ear rot [14] were evaluated after inoculation with conidial suspensions of *F. verticillioides*. Completely randomized block design experiments with three replicates were conducted in Pergamino, Province of Buenos Aires. Experimental units consisted of single 5-m rows sown at a rate of 5 plants per m. For inoculation, 2 ml of a conidial suspension (1×10^6^ conidia ml^−1^) were injected into the silk channel with a cattle vaccinator when silks exhibited brown tips, approximately 4 days after silking. A non-inoculated treatment for each inbred was also included. Fifteen ears of each inbred were manually harvested at 18–20% grain moisture and naturally dried to reach equilibrium moisture (nearly 12%). Disease severity was visually assessed in each plant as the ear area covered by mold (%). Percentages of disease severity were transformed into arcsin (percentage of disease severity)^1/2^ to normalize errors. The grains were stored in plastic bags (Ziploc®) at 4°C until biological analyses were carried out.

For silk assays, the same conidial suspension was injected into the silk channel, approximately 4 d after silking, covered with plastic bags and manually harvested 72 h after infection. Non-inoculated silks were sampled and used as control. Silks of fifteen plants of each inbred line were immediately frozen in liquid nitrogen and stored at −80°C.

### Ergosterol and Fumonisin Determination

Ergosterol was determined as an indicator of fungal biomass in grain. Ergosterol concentration was assessed following Iglesias et al. [1]. Samples (1 g) from contaminated milled grain were suspended in 15 ml of methanol for 2 minutes in a 125 ml erlenmeyer flask. The blend was poured into a 50 ml capped polypropylene centrifuge tube. The remaining blend from the Erlenmeyer flask was washed off with 15 ml of methanol, and poured into the centrifuge tube. The final extract was then centrifuged 15 min at 3000×g. The supernatant was poured off. The residue was re-suspended in 10 ml of methanol, shaken for 30 sec, and centrifuged as before. Supernatant portions were combined, mixed with 8.5 g of KOH and 25 ml of ethanol, and refluxed for 30 min at 65° C. The cooled, saponified mixture was diluted with 5 ml of distilled water and extracted three times with 10 ml of hexane. Hexane extracts were combined and evaporated to dryness under nitrogen with heating (35°C) in a rotatory evaporator. The resultant residue was dissolved in 5 ml methanol (HPLC grade). The solution was transferred to vials for HPLC analysis after filtration (0.22 µm). HPLC analysis was carried out with a Hewlett-Packard model 1050 system. Elution was performed at room temperature on a Hipersyl ODS C18 microbore column (200×2.1 mm, i.d. 5 µm) using an isocratic mobile phase consisting of methanol at a flow rate of 0.3 ml/min and detection at 282 nm. A volume of 10 µl was injected into the HPLC. Analyses were performed in duplicated and the results were obtained using the average.

Concentration of fumonisins in grain was assessed by ELISA (Ridascreens Fast Fumonisin, R-Biopharm AG, Darmstadt, Germany). Milled grain samples were thoroughly mixed, and two 5-g subsamples were taken. Fumonisins were extracted by blending each 5-g subsample in 25 ml of 70% methanol. The mix was shaken for 2 min in a vortex Boeco V-1 (Boeckel & Co., Hamburg, Germany), filtered through filter paper Whatman No. 1 and diluted 1∶14 with sterile distilled water. Diluted extracts were rediluted by adding a 5% aqueous solution of methanol with dilution factors of 1, 4, 20, 40, 60 and 150 for disease severity ratings of 1, 2, 3, 4, 5, 6 and 7, respectively. Diluted extracts and five standards, at concentrations of 0.000, 0.222, 0.667, 2.000 and 6.000 mg g^−1^ of fumonisins, were subjected to ELISA. Well absorbance was measured at 450 nm with a microplate reader Biotek 800 LX (Biotek Instruments Inc., Winooski, VT). Absorbance values of positive standards and samples were divided by the absorbance value of the first standard (standard zero). Concentration of fumonisins of the samples was estimated on the basis of a logit-log function between fumonisin concentration and relative absorbance of the four positive standards. RIDAs SOFT Win software (R-Biopharm AG, Darmstadt, Germany) was used for fumonisin determination.

### Scanning Electronic Microscopy (SEM)

Kernel colonization of pericarp and endosperm tissues from L4637 and L4674 was also evaluated by Environmental Scanning Electron Microscopy ESEM using QUANTA 200FEG (Field Emission Gun) microscope (FEI) from the Electronic Microscopy Laboratory of CCT Rosario (CONICET-UNR). The observation parameters were: energy: 7 KeV, working distance: 6 mm, humidity: 98%, temperature: 2°C and pressure: 8 mbar.

### Total RNA Extraction and RT-PCR

Grain samples from plots of each inoculated and non-inoculated inbred were milled in an IKA A11 basic Analytical mill. Total RNA was extracted from 0.5 g of milled grain and silk samples using the Trizol protocol (Invitrogen). RNA was then purified with the RNA Cleanup protocol (Qiagen) according to the manufacturer’s instructions. The amount and the quality of the total RNA were estimated in a Nanodrop spectrophotometer as well as by agarose gel electrophoresis.

cDNA was synthesized using 4 µg of total RNA and oligo(dT) with SuperscriptIII reverse transcriptase (Invitrogen), according to the manufacturer’s instructions and used for performing qRT-PCR.

### Microarray Hybridization

Maize oligonucleotide arrays service (Maize Oligonucleotide Array Project, University of Arizona, Tucson, AZ, USA http://ftp.maizegdb.org/MaizeGDB/FTP/arizona_maize_arrays) was used for the study. The array consists of 46,000 oligonucleotide probes, representing >30,000 identifiable unique maize genes. The probes were designed based on EST, cDNA, and genomic sequences mainly from the TIGR maize Gene Index, Consortium for Maize Genomics, the PLANT and dbEST divisions of GenBank, and individual investigators. Protocols used for cDNA labeling, hybridization and microarray scanning can be obtained in http://ftp.maizegdb.org/MaizeGDB/FTP/arizona_maize_arrays/.

### Experimental Design

**Figure 5:**
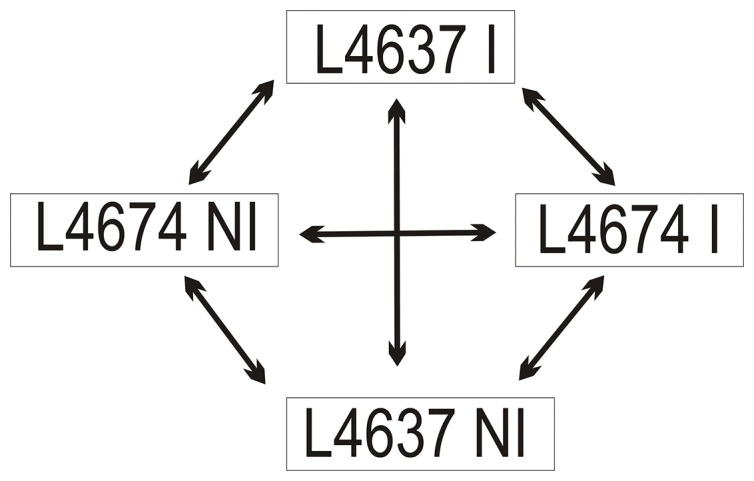
Experimental design of microarray experiment from grain tissues of two selected maize inbreds challenged by ***Fusarium verticillioides.***

Gene expression analysis was performed using RNA samples from non-inoculated (NI) and inoculated (I) inbred L4637 and L4674. Each experiment was carried out in three biological replicates and dye swaps. The experimental design of the transcriptional profiling is shown in Figure 5.

### Normalization and Data Analysis

The median foreground values for each channel were first normalized using the lowess method of the limma package in R (within each array) and then using limma’s quantile method (between all arrays) [45] . Probes with expression values higher than 3.0 standard deviations above the average foreground intensity of the negative controls in the arrays were included for the analysis. For differential expression, we used the unadjusted p-value generated by limma. Differentially expressed probes were identified using a 1.2 fold cutoff for expression ratios with a limma assigned p-value < 0.05.

Normalized data were log2 transformed and then fitted into mixed model ANOVAs [46], [47]. Estimates of the expression differences were calculated using the mixed model. Based on these statistical analyses, the spots with tests with an FDR less than or equal to 5% and with changes in signal intensity between each comparison of 1.2-fold or higher were considered as differentially expressed. Data were deposit in GEO, with an accession number GSE40288.

### qRT-PCR Expression Analysis

Gene expression data from microarray hybridizations were validated in maize grain samples by quantitative reverse transcriptase (qRT)-PCR analysis. This was performed on a subset of selected genes that were up- or down regulated during the infection in the L4637 and L4674 inbreds according to microarray analysis. The same set of genes was also tested in silk samples from both maize inbreds. Gene-specific primers were designed along the last exon and the 3′UTR region, using Primer3 software (http://frodo.wi.mit.edu/cgi-bin/primer3/prime3 www.cgi) and their sequences are shown in Table 3.

**Table 3:** Gene-specific primers used for qRT-PCR analysis.

Name	Identifier	maize sequence	Left primer	Right primer
**Glucanase**	MZ00030174	GRMZM2G125032	GCCTCTTCTACGGCAACAAG	AGTTAATTGCACCGCTCCTC
**Chitinase**	MZ00041277	GRMZM2G051943	CCTCGCGAAAAGTAAACCAA	CATGCTGCAAACGGAAAGTA
**Lipid-transfer protein**	MZ00003835	GRMZM2G320373	GTACAAGAGGAACCCCAGCA	GCCCAAAGGTGCTCAAGTAG
**26S proteosome**	MZ00041312	GRMZM2G121452	TGCAGCTTATCAACCAGACG	CAGATGAGAGCTGCGATCAA
**Xylanase inhibitor**	MZ00014433	GRMZM2G447795	ACAACAGGACCGGATACAGC	CATTCGATTATCACGAAAGTCTCTT
**Pathogen related protein**	MZ00014229	GRMZM2G112524	CATTGGACTGGGATGAGCTT	CCACACAGAAAACCATGACG
**Malic enzyme**	MZ00041285	GRMZM2G122479	CCAAGGGACCTGGTGAAATA	CTAGCAGCCACAAAGGTGGT
**Actin 1**	MZ00034191	GRMZM2G126010	CTTCGAATGCCCAGCAAT	CGGAGAATAGCATGAGGAAG

Twenty ng of single strand cDNA were used for qRT-PCR. SYBR Green PCR Master Mix (Invitrogen) was used for the PCRs according to the manufacturer’s protocol, with 2.5 mM MgCl_2_; 0.25 µM of each primer and 0.025 U Taq Platinum Polymerase (Invitrogen). Relative quantitative analysis was performed using a Stratagene device (Stratagene). Cycling parameters were as follows: initial denaturation at 95°C for 2 min; 45 cycles of 95°C for 10 s, and 58°C for 15 s; 72°C for 20 s. Melting curves for each PCR reaction were determined by measuring the decrease of fluorescence with increasing temperature (from 65°C to 95°C). The specificity of the PCR reactions was confirmed by melting curve analysis using the software as well as by agarose gel electrophoresis of the products. Relative quantification was normalized using *actin1* sequence (*MAc1*) [EMBL-EBI: J01238] as internal control and the expression ratio and FC were calculated using the 2−ΔΔCt method [48]. Each assay was run in triplicate and repeated at least three times using different samples.

### Metabolite Profiling

Metabolite analysis by Gas Chromatography–Mass Spectrometry (GC-MS) was carried out essentially as described by Lisec et al. [49]. Pools of different grain samples were ground in a ceramic mortar and pestle pre-cooled with liquid nitrogen and extracted with methanol. Ribitol was added as internal standard (0.3 mg ribitol mL^−1^ water MiliQ). The mixture was extracted for 15 min at 70°C and mixed with chloroform and water. After centrifugation at 2,200 *g*, an aliquot of 50 µl of supernatant was transferred to eppendorff tube and dried in vacuum concentrator. The samples were derivatized and GC-MS performed as described by Lisec et al. (2006). The mass spectra were cross-referenced with those in the Golm Metabolome Database [50]. Six independent determinations, composed by two lines and two treatments, were performed.

## Supporting Information

File S1 **Differentially expressed genes in L4637 vs L4674 after** ***Fusarium verticillioides*** **infection.**
(XLS)
